# Non-Dipper Blood Pressure Impact on Coronary Slow Flow in Hypertensive Patients With Normal Coronary Arteries

**DOI:** 10.7759/cureus.33356

**Published:** 2023-01-04

**Authors:** Kaya Özen, Mehmet Zülkif Karahan

**Affiliations:** 1 Cardiology, Gazi Yasargil Training and Research Hospital, Diyarbakır, TUR; 2 Cardiology, Mardin Artuklu University, Faculty of Medicine, Mardin, TUR

**Keywords:** non-dipper, timi frame count, dipper, coronary slow flow, hypertension

## Abstract

Objective: Coronary slow flow (CSF) is linked to myocardial ischemia, malignant arrhythmias, and cardiovascular mortality. On the other hand, hypertension (HTN) is an important risk factor for vascular disorders. There is limited research on the relationship between CSF and HTN. This study aimed to investigate TIMI frame count (TFC), which is an indicator of CSF, in dipper and non-dipper hypertensive individuals with normal coronary arteries.

Methods: The study was conducted as a retrospective observational study. Patients diagnosed with CSF and dipper or non-dipper hypertension were included in this study. Blood tests were routinely conducted for all patients. ECG was conducted for each patient, and echocardiography was performed. Coronary artery images were obtained in the CAG laboratory. Blood pressure (BP) measurements were obtained from the ambulatory Holter records. The patients were separated into two groups based on ambulatory Holter monitoring. The relationship between CSF and HTN was also examined.

Results: A total of 71 patients, comprising 25 women (37.2%) and 46 men (62.8%) with an average age of 52.75±9.42 years, were enrolled in the research. Based on ambulatory BP, the individuals were separated into two groups: non-dipper (n=36) and dipper (n=35). The pulse rate was significantly higher in the non-dipper group (p<0.001). In terms of mean systolic and diastolic blood pressure, there were no substantial differences across the groups (p = 0.326 and p = 0.654, respectively). The daytime mean systolic and diastolic BP did not significantly differ across the groups (p = 0.842 and p = 0.421). The dipper group had substantially lower nighttime systolic and diastolic BP values (p <0.001). The LAD, Cx, and RCA TIMI frame scores were significantly lower in the dipper group (p<0.001).

Conclusion: In this study, non-dipper patients had a greater CSF rate than dipper.

## Introduction

Coronary slow flow (CSF) is defined as prolonged opacification of the distal artery in the absence of substantial angiographic coronary obstruction [1]. Thrombolysis in myocardial infarction frame count (TFC) is the total number of ciné-angiographic frames collected following the pumping of an opaque substance into the coronaries, from the time the color appears at the coronary artery ostium to the distal portion [2]. A simple, objective, and repeatable, TFC provides a quantifiable indicator of coronary blood flow [3]. A higher TFC is indicative of diminished coronary blood flow.

CSF may generate angina-like chest pain during rest or exertion [4,5]. It has been associated with myocardial ischemia, life-threatening arrhythmias, and sudden cardiac death [6]. CSF's genesis is not yet been elucidated. On the other hand, hypertension is an important risk factor for myocardial infarction, stroke, and other vascular diseases [7,8].

Blood pressure (BP) is highest in the morning, gradually decreases over the day, and remains lowest at night. According to ambulatory BP monitoring, dipper hypertension is diagnosed when nighttime BP drops by more than 10% compared to daytime values, whereas non-dipper hypertension is diagnosed when the reduction is less than 10% [9]. Hypertension in non-dippers is associated with damage to organs such as the vascular system [10].

We aimed to investigate coronary slow flow in dipper and non-dipper hypertensive patients with normal coronary arteries.

## Materials and methods

Study design and subject

This retrospective study was conducted at a tertiary center between 2018 and 2022. Patients with chest pain who had normal coronary angiography and were diagnosed with arterial hypertension by ambulatory blood pressure monitoring were enrolled in this study. The CSF was determined by the AHA guidelines for coronary artery revascularization published in 2021 [11]. Seventy-one patients with arterial hypertension were enrolled in this research. Patients with coronary artery disorders, hyperthyroidism, hypothyroidism, mild or severe valve disease, heart failure, left ventricular hypertrophy, chronic renal failure, active cancer, chronic obstructive pulmonary disease, or congenital heart disease were excluded. Patients whose data were inaccessible and whose ambulatory blood pressure monitoring analysis was unsuccessful were excluded from this study. The local ethics committee (Gazi Yaşargil Training and Research Hospital) approved the study protocol (No: 2022-221). It adhered to the Declaration of Helsinki's ethical guidelines for human experimentation (Date: 25/11/2022) (2013).

Study protocol

Blood tests were routinely performed for all patients. ECG was conducted for each patient; and echocardiography was performed. The formula for calculating the body mass index (BMI) was weight divided by the square of height. Coronary artery images were obtained in the coronary angiography (CAG) laboratory. Blood pressure (BP) measurements were obtained from the ambulatory Holter records.

Definitions

The ambulatory blood pressure device (DMS 300-3A Holter Recorder) was worn by trained and experienced personnel. Every 15 minutes between 07:00 and 23:00, and every 20 minutes between 23:00 and 07:00, blood measurements were taken. Using small time intervals, the interval from 10:00 to 22:00 was considered daytime, while the span from 24:00 to 6:00 was considered nighttime. Non-dipper HTN was classified as reducing less than 10% or remaining unchanged in the mean systolic and diastolic BP levels. Dipper HTN was classified as reducing more than 10% in the mean systolic and diastolic BP levels.

The patients' coronary arteries were examined using the Philips Integris H 3000 angiography system (Eindhoven, The Netherlands). The TFC of individuals with normal coronary arteries was independently determined for each coronary artery. Nitrate was not supplied to any patient during CAG because it might have influenced the measurement findings. Coronary artery images were reviewed by two experts who were blinded to the research.

Statistics

IBM Corp. Released 2016. IBM SPSS Statistics for Windows, Version 24.0. Armonk, NY: IBM Corp. was used for the analysis. Initial continuous variables are represented as mean ± standard deviation or median (interquartile range). The normality distribution of the variables was analyzed utilizing the Kolmogorov-Smirnov and Shapiro-Wilk tests. Frequencies and percentages were used to represent categorical variables. The chi-squared test or Fisher’s exact test was utilized for categorical variables. Student’s t-test or Mann-Whitney U-test was utilized to compare continuous variables, as appropriate. If the p-value was less than 0.05, all tests were deemed statistically significant.

## Results

A total of 71 patients, comprising 25 women (37.2%) and 46 men (62.8%) with an average age of 52.75±8.22 years, were enrolled in the research. The patients' clinical characteristics were summarized in Table 1.

**Table 1:** Clinical characteristics of the patients Data are expressed as mean SD, number (percentage), or median (interquartile range) as appropriate. BMI: body mass index; FPG: fasting plasma glucose; HDL: high-density lipoprotein cholesterol; LDL: low-density lipoprotein cholesterol; TG: triglycerides; EF: ejection fraction; BB: beta-blocker; CCB: calcium channel blockers; ACEI: angiotensin-converting enzyme inhibitors; ARB: angiotensin receptor blocker

Parameters	Patients (n=71)
Age (years)	52.75±8.22
Gender, female, n (%)	25 (35.2)
BMI (kg/m^2^)	23.53±2.7
Smoking, n (%)	38 (53.5)
Diabetes mellitus, n (%)	9 (12.6)
Hyperlipidemia, n (%)	21 (29.6)
Hemoglobin (g/dl)	13.2±1.54
FPG (mg/dl)	95.78±13.8
Creatinine (mg/dl)	0.78±0.15
HDL-C (mg/dl)	46.76±12.34
LDL-C (mg/dl)	131.55±38.92
TG (mg/dl)	132.12±72.23
EF (%)	65.63±3.2
Antihypertensive drugs	
BB, n (%)	29 (40.8)
CCB, n (%)	25 (35.2)
ACEI, n (%)	22 (30.9)
ARB, n (%)	23 (32.4)

When their distribution according to disease states was examined, 12.6% had diabetes mellitus (DM), and 29.6% had hyperlipidemia. The smoking rate was found to be 53.5%. The mean BMI was 23.53±2.7, and the mean LVEF was 65.63±3.2. Beta-blockers were the most used anti-hypertensive drug, with a rate of 40.8%. Patients were separated into two groups based on ambulatory blood pressure: dipper (n=35) and non-dipper (n=36). There were no substantial differences in the hematological and biochemical parameters between the groups, as shown in Table 2.

**Table 2:** Clinical characteristics between the groups Data are expressed as mean SD, number (percentage), or median (interquartile range) as appropriate. BMI: body mass index; FPG: fasting plasma glucose; HDL: high-density lipoprotein cholesterol; LDL: low-density lipoprotein cholesterol; TG: triglycerides; EF: ejection fraction; BB: beta-blocker; CCB: calcium channel blockers; ACEI: angiotensin-converting enzyme inhibitors; ARB: angiotensin receptor blocker

PARAMETERS	Dipper N=35	Non-dipper N=36	P-Value
Age (Years)	51.62±9.2	54.2±3.6	0.286
Gender, female, n (%)	16 (45.7)	9 (25)	0.092
BMI (kg/m^2^)	22.79±2.81	24.47±2.92	0.032
Smoking, n (%)	16 (45.7)	22 (61.1)	0.294
Diabetes mellitus, n (%)	4 (11.2)	5 (13.8)	0.328
Hyperlipidemia, n (%)	10 (28.5)	11 (30.5)	0.910
Hemoglobin (g/dl)	13.10±1.72	13.42±1.41	0.721
FPG (mg/dl)	93.72±15.6	97.62±16.4	0.259
Creatinine (mg/dl)	0.80±0.16	0.76±0.18	0.408
HDL-C (mg/dl)	46.23±13.8	47.02±13.6	0.783
LDL-C (mg/dl)	133.55±17.8	131.56±17.2	0.687
TG (mg/dl)	114.50±59.7	149.83±81.2	0.069
EF (%)	65.50±4.2	65.8±4.5	0.539
Anti-hypertensive drugs			
BB, n (%)	14 (40)	15 (41.6)	0.906
CCB, n (%)	11 (31.4)	14 (38.8)	0.114
ACEI, n (%)	12 (34.2)	10 (27.7)	0.324
ARB, n (%)	10 (28.5)	13 (36.1)	0.086

The BMI was substantially higher in the non-dipper group (p=0.032). Patients' 24-hour ambulatory blood pressure results were analyzed and evaluated (Table 3).

**Table 3:** Comparative analysis of ambulatory blood pressure between groups

Ambulatory blood pressure	Dipper N=35	Non-dipper N=36	P-Value
Pulse (bpm)	67.47±4.82	72.68±4.92	<0.001
Over 24-hours			
Mean systolic blood pressure (mmHg)	118.87±13.8	124.27±18.23	0.326
Mean diastolic blood pressure (mmHg)	67.89±9.55	69.47±7.64	0.654
Day-time			
Mean systolic blood pressure (mmHg)	122.37±16.78	124.54±18.08	0.842
Mean diastolic blood pressure (mmHg)	72.64±10.49	69.78±8.45	0.421
Night-time			
Mean systolic blood pressure (mmHg)	108.8±12.58	123.48±18.32	<0.001
Mean diastolic blood pressure (mmHg)	60.40±7.84	69.12±8.1	<0.001
Systolic blood pressure variation (%)	13.57±3.47	1.02±3.49	<0.001
Diastolic blood pressure variation (%)	15.24±4.73	2.01±4.03	<0.001

The pulse rate was significantly higher in the non-dipper group (p<0.001). In terms of mean systolic and diastolic BP, there were no substantial differences across the groups (p = 0.326 and p = 0.654, respectively). The daytime mean systolic and diastolic BP did not significantly differ across the groups (p = 0.842 and p = 0.421, respectively). The dipper group had substantially lower nighttime systolic and diastolic BP values (p <0.001). The variations in systolic and diastolic BP were substantially higher among dippers compared to non-dippers (p <0.001). The mean TIMI frame score of the coronary arteries was calculated (Table 4).

**Table 4:** Comparison of group scores on the TIMI frame Data are expressed as mean ± SD. LAD: left anterior descending artery; Cx: circumflex artery; RCA: right coronary artery; TIMI: thrombolysis in myocardial infarction

TIMI frame scores	Dipper N=35	Non-dipper N=36	P-Value
LAD TIMI frame score	34.18±2.68	38.07±3.45	<0.001
Cx TIMI frame score	21.28±3.24	25.63±2.64	<0.001
RCA TIMI frame score	15.78±2.70	20.53±2.48	<0.001
The mean TIMI frame score	19.87±4.21	23.87±2.59	<0.001

The left anterior descending artery (LAD), circumflex artery (Cx), and right coronary artery (RCA) TIMI frame scores were substantially lower in the dipper group (p<0.001).

## Discussion

The findings of the research revealed that TFC, which is an indicator of CSF, was higher in non-dipper hypertensive patients. The LAD, Cx, and RCA TIMI frame scores were significantly lower in the dipper group.

Coronary circulation is typically observed in two compartments. The first compartment contains epicardial vessels, known as conducting vessels owing to their lack of resistance to blood flow. The second compartment is composed of the microvascular vessel, known as the resistance vessel, that controls myocardial blood flow [12]. Microvascular coronary vessel dysfunction is responsible for CSF [13]. In cardiovascular and cerebrovascular patients, decreased endothelium-dependent vasodilation due to a reduction in nitric oxide release in the microvascular arteries is a risk factor [14]. Karakaya et al. observed that the velocity of cerebral blood flow was greatly reduced in individuals with CSF [15]. Endothelial irregularity appears to be a systemic condition that affects both the coronary and peripheral arteries. Günlü et al. found a correlation between carotid intima-media thickness and endothelial dysfunction in hypertensive patients [16]. Higashi et al. evaluated endothelial dysfunction in 20 hypertensive patients with and without dippers [17]. In their study, the endothelial dysfunction indicators nitrite/nitrate and cyclic guanosine monophosphate were substantially lower in the non-dipper patient group.

Gibson et al. suggested TFC as a straightforward and quantitative method for producing a standard coronary blood flow assessment indicator by analyzing angiographic data from the TIMI-4 trial [18]. A high number of TIMI frames are associated with sluggish blood flow and endothelial dysfunction. Aksit et al. stated that patients with CSF had a higher proportion of non-dipper patients than dipper patients [19]. Additionally, sudden cardiac death and the frequency of malignant arrhythmia were higher in the non-dipper group with normal coronary arteries.

In a study by Xu et al., TIMI scores were higher in the hypertension group than in the normotensive group [20]. When patients with normal coronary arteries were compared for positive or negative myocardial perfusion scintigraphy, patients with positive scintigraphy had substantially greater TFC. Based on these findings, they hypothesized that coronary artery flow and myocardial perfusion abnormalities would be more prevalent in the group with high TFC. They emphasized that myocardial perfusion scintigraphy could be employed as a noninvasive method for detecting slight changes in cardiac microcirculation. If we take into consideration the TFC as an indicator of endothelial dysfunction in CSF, our study results, such as a higher TFC in the coronary arteries, confirm the results of previous studies.

Crea et al. reported that platelet count, high-sensitivity C-reactive protein, and serum uric acid were higher in hypertensive patients with CSF than in the control group [21]. In recent studies, the TFC was substantially higher in patients with metabolic syndrome than in those without metabolic syndrome, with greater BMI values [22,23]. In our research, the non-dipper group had a significantly higher BMI than the dipper group.

Limitations

The study population was small. Tests for other biochemical and echocardiographic markers showing an association with the slow coronary flow were not performed. The diameters of the coronary arteries did not match. Vasoconstriction, which may result from an elevation in sympathetic tone, may have influenced the TIMI frame counts.

## Conclusions

In this study, TFC, an indicator of CSF, was observed to be higher in non-dipper hypertensive patients who underwent coronary angiography. Previous studies have shown that endothelial dysfunction, subclinical atherosclerosis, and inflammation are related to the occurrence of CSF. Our findings support that CSF may be part of systemic vascular disturbance. Considering that this situation can lead to fatal arrhythmia and sudden death, non-dipper hypertensive patients should be followed up more frequently and treated effectively. Further clinical studies are needed to examine the relationship between non-dipper HTN and CSF.
